# Structural coercion in the context of community engagement in global health research conducted in a low resource setting in Africa

**DOI:** 10.1186/s12910-020-00530-1

**Published:** 2020-09-21

**Authors:** Deborah Nyirenda, Salla Sariola, Patricia Kingori, Bertie Squire, Chiwoza Bandawe, Michael Parker, Nicola Desmond

**Affiliations:** 1 Malawi Liverpool Wellcome Trust Clinical Research Programme, PO Box 30096, Chichiri, Blantyre 3, Malawi; 2 Department of Social Sciences, University of Helsinki, P.O 18 (Unioninkatu 35), 00014 Helsinki, Finland; 3 The Ethox Centre/ Wellcome Centre for Ethics and Humanities, University of Oxford, Oxford, UK; 4 University of Malawi College of Medicine, Private Bag 360, Chichiri, Blantyre 3, Malawi; 5 Liverpool School of Tropical Medicine, Pembroke Place, Liverpool, L3 5QA UK

**Keywords:** Global health, Community engagement, Health research, Structural coercion, Research ethics, Bioethics, Africa

## Abstract

**Background:**

While community engagement is increasingly promoted in global health research to improve ethical research practice, it can sometimes coerce participation and thereby compromise ethical research. This paper seeks to discuss some of the ethical issues arising from community engagement in a low resource setting.

**Methods:**

A qualitative study design focusing on the engagement activities of three biomedical research projects as ethnographic case studies was used to gain in-depth understanding of community engagement as experienced by multiple stakeholders in Malawi. Data was collected through participant observation, 43 In-depth interviews and 17 focus group discussions with community leaders, research staff, community members and research participants. Thematic analysis was used to analyse and interpret the findings.

**Results:**

The results showed that structural coercion arose due to an interplay of factors pertaining to social-economic context, study design and power relations among research stakeholders. The involvement of community leaders, government stakeholders, and power inequalities among research stakeholders affected some participants’ ability to make autonomous decisions about research participation. These results have been presented under the themes of perception of research as development, research participants’ motivation to access individual benefits, the power of vernacular translations to influence research participation, and coercive power of leaders.

**Conclusion:**

The study identified ethical issues in community engagement practices pertaining to structural coercion. We conclude that community engagement alone did not address underlying structural inequalities to ensure adequate protection of communities. These results raise important questions on how to balance between engaging communities to improve research participation and ensure that informed consent is voluntarily given.

## Background

Community engagement activities are increasingly expected in global health to address power and health inequities between those designing and implementing research and target groups. For much of the twentieth century, research communities were openly regarded as passive research subjects but more recently health movements across the world began demanding opportunities to influence research objectives and participate actively in knowledge production [1]. For example, in the US and Europe from the 1970s and 80s, women’s health advocates and communities affected by HIV/AIDS demanded involvement in designing research on contraceptives and antiretroviral drugs respectively [1, 2]. Simultaneously, participatory approaches were increasingly viewed as crucial for effective and ethical conduct of development programmes in the global south [3]. In response, engaging communities in global health research became an ethical expectation and a requirement of most donors [1].

While core ethical principles for human subject protection have traditionally focused on the rights of individual research participants over community interests, some have argued that this is a limited, solipsistic view, recommending a broader community-based focus on research participation [4]. The basis of this argument is that decisions are embedded within social networks and that some individuals rely on other people to make decisions on research participation [5]. In addition, outcomes of some biomedical research are likely to impact communities and not solely research participants [4]. In recognition of these arguments, recommendations to promote community engagement in health research have been incorporated in a wide range of ethical guidelines including those from the Council for International Organisations of Medical Sciences (CIOMS) which states that:

*'...researchers, sponsors, health authorities and relevant institutions should engage potential participants and communities in a meaningful participatory process that involves them in an early and sustained manner in the design, development and implementation, implementation of informed consent processes and monitoring of research and in the dissemination of its result*s' [6].

Community engagement is also said to enhance protection of research participants and non-research participants, minimize risks, enhance benefits and legitimacy of research projects [3]. Moreover, it is considered as an important strategy to resolve power differences between researchers and communities by allowing marginalised voices to be included in the production of scientific knowledge.

Despite the attention given to community engagement in international ethical guidelines, few publications have focused on understanding whether and in what ways community engagement, in practice, improves the ethical conduct of research in the context of structural inequalities. Furthermore, attempts to prevent coercion and undue inducement in current ethical guidelines focus on individual, rather than broader social factors that may shape research participation. This paper seeks to address this omission by drawing on the literature on structural coercion to support empirical findings of structural forms of coercion arising in the context of community engagement in biomedical research conducted in Malawi.

### Structural coercion

The term ‘structural coercion’ is informed by the work of Paul Farmer (2006) on ‘structural violence’ which refers to how social arrangements can harm individuals and populations. He argues that ‘*the arrangements are structural because they are embedded in the political and economic organization of our social world and they are violent because they cause injury to people’* [7, 8]. For instance, an individual’s susceptibility to HIV infection and poor outcomes may be due to social factors such as poverty, gender inequality as well as limited access to post-exposure prophylaxis [8]. A lack of attention to these forms of structural violence may limit the effectiveness of medical interventions, or worse, create conditions that capitalize on them. Based on this concept of structural violence, Fisher (2013) introduced the term structural coercion to describe how ‘*the broader social, economic and political context compels individuals to enrol in research’* [9]. For instance, people with limited access to medical treatment may feel compelled to enrol in clinical trials to access treatment and not primarily to advance science [10]. Structural coercion could therefore be avoided by paying attention to the socio-economic context and power dynamics between researchers and participants. Drawing on these arguments, Kingori (2013) presents the concept of ‘empty choice’ as a critique of the current overemphasis on individual choice as the dominant marker of ethical research in contexts where research participation represents the most viable option for the poor to access income and health services [11]. This paper extends arguments of structural coercion to ethnographic research examining community engagement in three research projects implemented in Malawi. We demonstrate that, contrary to literature that presents community engagement as the solution for structural violence and coercion, community engagement practices may in some cases create opportunities for structural coercion and thus adversely affect the ethical conduct of research.

## Methods

### Study setting

The ethnographic data informing this paper was collected in Blantyre and Chikwawa districts of southern Malawi, where a large number of research projects are implemented. Malawi is located in the southern part of Africa with a total population of 17,215,000, a majority of whom (84%) reside in rural areas [12]. There are 28 districts in Malawi which are further sub divided into wards and villages headed by village headmen or traditional chiefs. These traditional chiefs are responsible for settling disputes, mobilising local labour for development initiatives as well as to advance the government’s agenda in the communities. Despite becoming a democratic state in 1994, Malawi’s democracy has been critiqued as having hierarchical structures of social relations as well as strong authoritarian strains [13]. In Malawi, over 72% of the population live below the poverty line of less than USD 1.25 a day [14]. The literacy rate is 65% [15] however the level of scientific literacy is much lower. Some of the leading determinants of poor health outcomes are low levels of education and poverty. There are many biomedical research projects implemented by both Malawian and non-Malawian researchers and this is evident in a growing list of scientific publications from 100 to 400 publications between 1995 and 2010 [16]. Despite its small population size compared to most African countries, Malawi is ranked as the 15th country in Africa with the highest scientific publication outputs in international journals [16], highlighting the intensity of scientific research projects conducted.

### Study design

This research aimed at gaining in-depth understanding of biomedical research community engagement activities, as experienced by different stakeholders such as researchers, field workers, community leaders, research participants, non-research participants, community volunteers and other research staff. To achieve this aim, three biomedical research projects from different institutions in urban, rural and hospital settings (undisclosed for anonymity purposes) were purposively selected for geographical variation as ethnographic case studies. The three research projects will be presented as the urban, rural and hospital case studies throughout the paper as presented in Table 1. DN spent a period of three months in the sites where research was being conducted to become familiar with the context, participate in community engagement activities and observe interactions between research stakeholders. Forty-three In-depth Interviews (IDI) and 17 Focus Group Discussions (FGD) were conducted with research participants, non-research participants, research staff, community leaders, community volunteers to understand experiences of community engagement and to seek explanations for some of the issues observed. Topic guides developed specifically for this study were used to interview different stakeholder groups and included topics such as: rationale for community engagement, experiences with community engagement, communication, perceptions of community engagement, motivations to participate in research, expectations on how they wish to be engaged, concerns with research and many more.

**Table 1:** Details of the case studies

	Urban case study	Rural case study	Hospital case study
Study design	Observational study	Cluster Randomised Trial	Longitudinal cohort study
Setting	Urban setting	Rural setting	Hospital setting
Target population	School communities School children	Villages All households in selected villages	TB patients
CE activities	Meetings with Primary Education Advisors Meetings with Parents and Teacher Association committees Meetings with parents Meetings with students Study information sent to communities	Community involvement to select volunteers and committees Training of community volunteers Village workshops facilitated by community volunteers Community volunteers involved in monitoring and evaluation	Consultation FGD Sensitization of health care workers
Aims of CE	Raise awareness about the study To get feedback on the research and engage in two-way dialogue with communities	To educate and empower communities to implement interventions aimed at preventing malaria	To explore patients and community members’ understanding of study information and to seek their feedback

### Summary of community engagement activities within the case studies

The nature and variation of community engagement activities across the three case studies shows how community engagement was conceptualised by the researchers to improve informed participation in research. In the urban case study, community engagement activities as defined by the research team included a meeting with senior education officials, followed by a series of meetings with parents and teacher committees, as well as students in all participating school communities. In the rural case study, community engagement activities as defined by the research team included the involvement of community members in selecting village committees and research volunteers, training of committee members and community volunteers on various aspects of community based interventions, weekly village workshops facilitated by community volunteers on health promotion and community involvement in implementing community and household level interventions. In the hospital based case study, community engagement as defined by the researchers involved consultation FGDs with potential study participants before implementing the study to seek feedback on the study as well as the study information sheets. A meeting was also organised by the research team with health care workers (HCWs) at the study site to communicate study details prior to implementation.

Most of the meetings in all three case studies were facilitated by research nurses and field workers to communicate study details before study implementation, except for the rural case study where some community members were engaged as peer educators throughout study implementation. In some cases, drama and songs were used to communicate study information in the urban and rural case study and an opportunity was often given to attendees to ask questions or seek clarification about the study. Community engagement meetings were often held in selected government primary schools, hospital buildings or open spaces at the study sites, and attendees ranged from 10 to 400 people. Common to all the community engagement activities were the involvement of district level government and non-governmental stakeholders such as teachers and health care workers as well as community leaders during community meetings or study implementation. Details of the research projects and community engagement activities employed are presented in Table 1.

### Data analysis

**Fig. 1:**
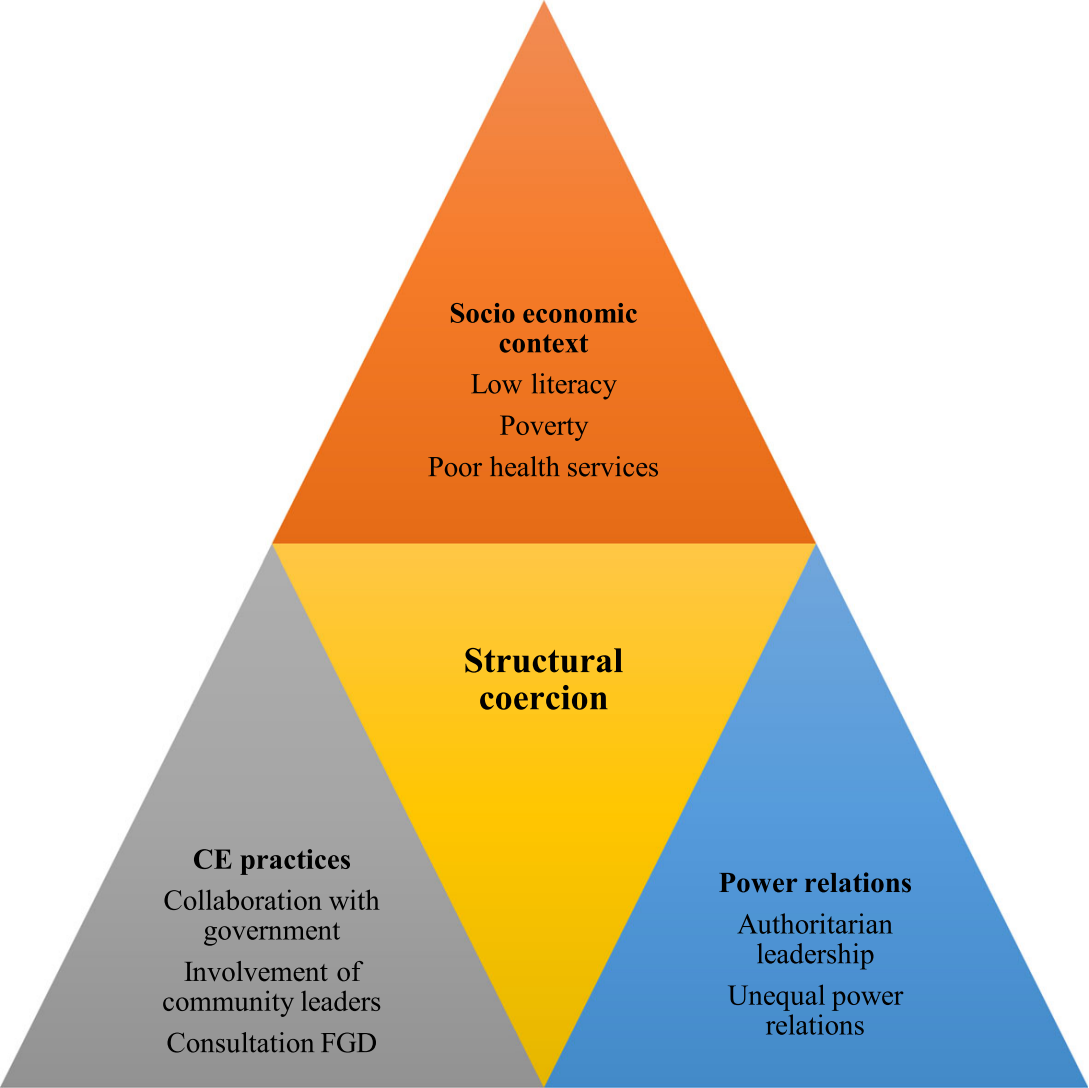
Structural coercion created through the interplay of key themes

All the IDIs and FGDs undertaken for this study were facilitated and recorded by DN. Experienced transcribers who were not involved in any of the case studies, transcribed the audio recordings into Chichewa. The first few transcripts from the IDI, FGD and field notes were read by DN to develop codes emerging from the data. A coding framework was developed in QSR Nvivo 10 and discussed with authors SS and ND during the coding process. Some of the initial themes included: aims of engagement, understanding of research, expectations from research, motivations to enrol in research, perceptions of research/community engagement, concerns with research and social relationships. Data collection, coding and analysis were ongoing and iterative such that emerging themes were further explored across the different research stakeholder groups. Thematic analysis was used to analyse the findings by describing the results in relation to a particular theme, comparing responses across respondents within the same case study and across case studies where relevant. Framework matrices were developed to compare responses among respondents in relation to a particular theme. Further analysis explored these themes in light of broader ethnographic observations about the socioeconomic context, community engagement practices and power relations between research stakeholders. The higher order theme that emerged from this analysis was structural coercion as shown in Fig. 1. Data was triangulated using multiple data collection methods, involving multiple sources of information and having multiple people (ND and SS) check and comment on the data analysis and interpretation. Explanatory and analytical accounts were based on explicit reasons given by the participants, implicit reasons inferred by putting together evidence and comparing findings with other studies. Preliminary descriptive findings were fed back to research teams and research participants for verification and validation.

### Ethics approval

This study was approved by University of Malawi, College of Medicine Research Ethics Committee and Liverpool School of Tropical Medicine Research Ethics Committee in the United Kingdom. We also sought approval to conduct the study from the directors of research institutions, study coordinators and village heads prior to data collection. Written consent was sought on an individual basis from all participants.

### Findings

The results highlight how an interplay of factors presented in Fig. 1 in relation to the social economic context, community engagement practices and power relations facilitated structural coercion. This complex interplay of factors is presented under the themes of perceptions of research as development, the power of translated medical research terminologies in influencing research participation, research participants motivation to access individual benefits and coercive power of community leaders.

### Perception of research as part of community development in community based studies

While guidelines on community engagement promote collaborations between researchers and stakeholders such as government agencies and non-governmental actors [6], such collaborations may influence research participation and undermine ethical practice.

As part of community engagement activities, all community-based research projects had links with local government. In the urban case study, researchers sought approval from the District Education Office before hosting community engagement meetings with parents and teacher committees; this gave the impression to some that the government had endorsed the study: *‘since you (the research team) have indicated that the Ministry of Education and College of Medicine (ethics review committee) have already accepted (approved) this study, we will give you support to implement the study’* (Head teacher, urban setting). Similarly, in the rural case study, researchers worked with HCWs employed by government to distribute bed nets, which led some people to attribute all research activities to collaboration with *aboma* [government]. Since most interventions run by the government, including vaccinations, are mandatory, some individuals participated in research with assumptions that research was also mandatory as shown in the quote below: *‘When researchers approach us, one cannot refuse. You just agree so that things should progress according to the government’s intentions.’* (FGD participant, rural setting-FGD012).

In most cases, the government and external service providers were viewed by citizens as powerful actors who acted in the best interests of the community to facilitate development and improve welfare. For instance, local government and other service providers constructed schools, hospitals and public roads, and were thus seen as instrumental in community development. In addition, a common saying during community engagement meetings was that *mlendo amadza ndi kalumo kokuthwa* [literal translation: a visitor brings a sharp knife], which meant that outsiders came with new knowledge to make positive contribution. During community meetings, conducted as part of community engagement activities, community leaders often described research as *chitukuko* [development] and thanked researchers for including their villages as part of such programmes. This suggests that the broader socio-economic context of poverty and the need for development assistance compelled some to think that their participation in research will lead directly to community benefits. Engagement of government stakeholders helped to gain buy-in for research, but also created the appearance that what was being implemented was a government programme, rather than biomedical research.

In addition, many research participants, including village leaders, had difficulties in differentiating between biomedical research and health services offered by Non-Governmental Organisations (NGO). During FGDs, participants were asked to state the research projects taking place in their villages. Responses included NGOs working in the communities such as: Hunger Project, Concern Universal and World Vision. Both research institutions and NGOs were often referred to as *mabungwe* [organisations] and community members often confused the different data collection goals of research and NGOs. Such confusion between research and non-research activities of NGOs and historical experiences of passively receiving aid caused some research participants to state that their participation was based on expectations of receiving support or facilitating development for their area. Thus, broader socio-economic disadvantages compelled some people to enrol in research with expectations of receiving economic support, without fully understanding either the specific research objectives or the risks involved. The information relayed during engagement activities focused on improving community acceptability of research and did little to minimize these misunderstandings.

### Research participants’ motivation to access individual benefits

Our research also showed that some research participants were motivated to participate in research to access clinical assessment, treatment and other forms of compensation. Health services in government facilities were offered free of charge to the general public. Most research participants in these settings, however, highlighted shortages of drugs, equipment and staff, leading to long waiting hours in public facilities. FGD participants in urban settings also indicated that some of the hospital based research projects were providing quality health services (ancillary care) to individual research participants and their families, over and above standard health services and this led to preference for biomedical research. As such, some research participants, particularly in hospital-based studies, consented to participate in research to access better clinical assessment, individual test results and treatment. Given this, it was not surprising that when research projects hosted at health facilities offered clinical assessment, treatment, transport reimbursement and other material forms of compensation, individuals participated in order to access these benefits as shown below:

*'Research helps in various ways...you know a lot about your health, and it helps you to access treatment...there is no reason why one should refuse to participate in research...um! People refuse [to participate] out of ignorance...when you are sick they assist you, the assistance you get is better than for someone who is not participating in research'* (Mother of a research participant, urban setting- IDI 016)

*'It's true, some researchers bring their research projects and give out, let's say two tablets of Ufresh [laundry soap that costs less than 20 cents]. When some people hear that there is a research project and they are giving out soap, they rush...they rush to receive the soap, yes!'*

(FGD participant, urban setting-FGD 001)

In the first quote, a mother to a paediatric research participant emphasizes that she saw research as beneficial because it allowed her to access better clinical assessment and treatment. In the second quote, a woman also explains that some people participate in research because of perceived benefits or compensation offered. These quotes highlight the disconnect between arguments of researchers stating there is no undue influence in the provision of health care and other forms of minimal compensation for research participation and the influence to enrol to access these benefits amongst economically disadvantaged groups. Decisions to participate in research were not always due to informed understanding of the research but rather to negotiate underlying socio-economic needs. This suggests a level of structural coercion, community engagement activities failed to prevent participants from being unduly influenced to participate in research to access individual benefits as opposed to intended public health benefits. In addition, we observed that a majority of community members had limited understanding of research, but community engagement activities did not help to improve understanding of research or address therapeutic misconceptions of research. Rather, such therapeutic misconceptions enhanced research participation and thereby structurally coercing research participants.

Information about research compensation and other benefits was often relayed at community engagement meetings and circulated in the community. Such events fell short of meeting the ethical expectations of engagement to improve informed participation in research because some community members formed their own interpretations of research benefits and they participated in research to access these. Rather in the socio-economic conditions under which research was conducted, the major effect of community engagement was to enhance recruitment.

### The power of vernacular translations of medical research terminologies in influencing research participation

Vernacular translation of some biomedical research terminologies during community meetings in addition to involvement of HCWs also contributed to therapeutic misconceptions and thereby influenced research participation. During community engagement events, research was translated as *‘kafukufuku’* in the vernacular language which means ‘finding out’. Data from the IDIs and FGDs also showed that biomedical researchers were interchangeably referred to as *akafukufuku* [researchers], *achipatala* [people from the hospital] or *azaumoyo* [community health workers]*.* In practice, most ‘biomedical researchers’ involved in the case studies were HCWs and the research was either conducted in health facilities or involved clinical procedures and biomedical interventions. This meant that many community members had challenges in differentiating between biomedical research and clinical assessments as shown in the following quote: *“There are a lot of people who are sick out there but they just don’t know what is wrong with them...but if you come here and join research, they screen you and give you assistance (treatment) until you get well...”* (Research participant, hospital setting, IDI030).

In addition, vernacular translation of other research terminologies during community engagement meetings also played a role in revering biomedicine as more advanced to address health problems and influenced research participation. Indigenous healing practices *[mankhwala a chi****kuda or*** *zitsamba]* offered by traditional healers were generally discouraged by HCWs while biomedicine *[mankhwala a chi****zungu*** *or chipatala]* was promoted as safer. Interestingly, the word ‘*chi****kuda***’ is derived from the word -***kuda*** which means darkness, dirt or black; while the word ‘*chi****zungu***’ is derived from the word *m****zungu*** which means white, European or modern. In the settings where research was conducted, many research participants had low levels of literacy and viewed health care workers as experts with biomedical knowledge on how to address their health problems, regardless of their actual expertise. Since they felt that those providing research services were biomedical experts, participants felt they needed to follow instructions from biomedical researchers unquestionably since they knew the causes and cures of disease. For example, one FGD participant from an urban setting said: ‘*Malaria comes through various ways, we do not know how malaria comes about. The researchers [health workers] know that malaria comes in this way and for us to prevent or eradicate malaria we need to do this and that...’* (Female research participant, urban setting-IDI 015 ND). Such perceptions of health care workers as custodians of biomedicine, considered more advanced, obliged some community members to participate in biomedical research.

### The coercive power of community leaders in community based research

While we have shown examples of how structural coercion manifested in the conduct of biomedical research; IDIs, FGDs as well as participant observations also revealed cases where research participants were coerced to participate in research due to authoritarian leadership structures and unequal power relations within the community. Engagement of community leaders in research is considered as culturally appropriate in most African settings and a common engagement practice since it encourages participation and demonstrates respect towards community structures. It was however clear in this study that some community leaders, particularly in the rural setting, employed traditional authoritarian power structures to facilitate participation in research. This consequently undermined an individual’s autonomy to make decisions to participate in research voluntarily.

In the rural case study, researchers involved village leaders in community engagement meetings and other research activities as a way of respecting cultural norms. During participant observation, it became apparent that village leaders sometimes enacted and enforced certain bylaws by using threats, some village leaders also applied similar approaches to foster research participation. For instance, some of the village leaders threatened people that they will be thrown out of the village or that they will be restricted from accessing social services to ensure compliance with research activities. This led some community members to comply with research procedures out of fear of consequences:

*'Of course, there were a few challenges, but the chief used his power, and everyone complied...he was telling people that their mosquitoes would infect others and if they suffer from malaria, they should not go to the health facility. So, everyone complied...'* (FGD participant, rural setting-FGD012).

*'Yes, I call for the meetings by myself...I tell them ‘if you don't come to this meeting, I will not write down your name for other relief items'* (Community leader, IDI024, rural setting).

The normalised use of threats among some village leaders and villagers’ fear of consequences for non-compliance therefore coerced some people to participate in research and compromised ethical research practice; often without the knowledge of the researchers. In addition, community acceptability of health interventions was higher in villages where research volunteers had support from village leaders. Attaining high coverage rates was necessary for research purposes to prevent malaria but also desirable when reporting progress during monthly meetings so the research team rarely questioned the ethics of the community leaders influence. This example highlights conflicts between international research ethics and socially accepted norms of authoritarian leadership to promote participation in public health interventions. In addition, it demonstrates researcher’s challenges to balance the need to engage communities to improve informed participation with the need to ensure voluntary participation in research. Even though engagement of community leaders is recommended and a common practice in global research, in such authoritarian settings, the use of threats to influence participation in research is disempowering to individual autonomy. In this case, engagement of community leaders did not effectively protect communities from being compelled to participate in research, rather it actively promoted coercion.

## Discussion

Overall, our results suggest that the inclusion of a standard community engagement approach alone is insufficient to empower research participants to make informed choices about research participation due to underlying structural and power inequalities. The three biomedical research projects were conducted in socio-economic contexts with low levels of literacy and poverty as well as poor health services. Poverty and low literacy levels are some of the socio determinants of poor health that make the conduct of bio-medical research rational to improve health. On the other hand, the same factors that provide justification to conduct biomedical research when combined with unequal power relations also increase vulnerability to exploitation. We have demonstrated how these inequalities led to structural coercion in the context of community engagement in a low resource setting.

Even though some studies have shown that engaging local stakeholders such as community leaders is essential for successful research implementation in low resource settings [17, 18], findings from this study support previous contradictory research that this can also compromise individual autonomy to consent to participate [18, 19]. While it is socially acceptable for community leaders to exercise their authority to ensure that communities comply with public health interventions, this may translate to coercive agreement to enrol in research due to threats from community leaders. Our study corroborates Angwenyi’s findings in Kenya that engagement of community leaders to improve informed participation resulted in coercion [19]. Community engagement therefore created opportunities for community leaders to exercise their power and influence individuals to consent in research and thereby compromised the ethical conduct of research.

The researchers’ aim of conducting research inadvertently reinforces views that portray biomedical research as legitimate to provide solutions to address community health concerns [20]. In addition, translations of some biomedical terminologies into vernacular as well as the involvement of health care workers also influence some people to participate in research with intentions of accessing clinical care and other benefits*.* Existing scholarship in this area also shows that power inequalities due to poverty, illiteracy and limited access to health services present high risks of exploitation in many low resource settings [21]. This complex interplay between the socioeconomic context and unequal power relations lead some community members to participate in research with expectations of benefiting from community development, medical treatment and other individual benefits. Community engagement meetings thus serve as the harbinger of these raised expectations*.* Our study contributes to the literature on community engagement and bioethics by showing how these factors may both enhance participation and seeming trust in research, while at the same time facilitate structural coercion and limit true voluntary participation in research. Long term participatory engagement of communities throughout research design and implementation may help to understand and address these contextual factors in order to avert structural coercion.

Balancing between employing community engagement to ensure voluntary participation in research and recruiting study participants to reach a desired sample size, within a limited time period is a contradiction that remains to be addressed in community engagement. While we agree that principles of research ethics should allow for voluntary participation in research [6], community members’ participation is also desirable in order to reach optimum sample size and demonstrate statistical power. On the other hand, community members’ refusal to participate in research or health interventions could potentially affect the statistical power of the study and eventually deprive the same communities of public health interventions that may improve health or delivery of health services. Our study has shown that some of the enabling factors that promoted research participation in some villages were likely due to the coercive power of local leaders, involvement of government stakeholders and perceptions or misperceptions of research benefits. In addition, community lack of awareness of research ethics as well as misunderstanding between biomedical research, clinical care and community development led to vulnerability to structurally coercive enrolment of research participants. Measuring the success of community engagement based on enrolment may therefore mask underlying unethical factors that actually enhanced research participation. We concur with Pickersgill (2007) that efforts need to be put in place to adhere to ethical guidelines and improve community understanding of research ethics to enhance their ability to assess risks of research and minimize exploitation [22]. Further research is also required to generate evidence on context relevant community engagement approaches that may enhance informed participation in research, particularly in low literacy settings.

From the existing scholarship in this area, there are some contributions which argue that community engagement improved informed participation and ethical research practice [23, 24]. In cases where community engagement was reported to improve informed consent, Boga (2011) for instance, reported that they engaged with communities to develop context relevant consent forms for different study designs in Kenya. This exercise helped to improve community understanding of study information before giving consent [24]. Ethical issues pertaining to community engagement have however been widely discussed particularly in the literature on Community Advisory Boards (CABs) [25–29]. Nevertheless, successful examples of engaging CABs to enhance ethical conduct of research have been reported in cases where CABs have been properly set up, they understand their advisory roles and they have the ability to analyze and communicate ethical issues [27]. Structural coercion could similarly be minimized by developing CABs who are empowered to analyze ethical issues pertaining to study implementation and advise researchers accordingly.

One of our study limitations is that we were unable to explore community’s perspectives on how structural coercion could be minimised in the conduct of global biomedical research because the study was not designed to seek community views on recommendations. We however engaged various research stakeholders at an ethics workshop to discuss ways of strengthening ethical community engagement in Malawi, and this has been published elsewhere [30]. Workshop discussions focused on enhancing collaborations between international and local researchers, consulting pre-existing stakeholder groups such as District Health Committees for input on research design, and improving communication with participating research communities using multiple communication channels. These workshops were however attended by researchers from various research institutes as well as ethics review committee members and therefore excluded views of community stakeholders. Future research aims to explore community perspectives on how to enhance ethical community engagement.

## Conclusion

This paper highlights how broader socio-economic factors invite potential for structural coercion in this low resource setting, despite the best intentions of those who employ community engagement approaches for ethical reasons. Given this, standardised community engagement does not adequately protect communities from being unduly influenced to participate in research. Rather, broader socio-economic contexts and unequal power relations inadvertently facilitate ‘unethical’ research participation. These results raise important questions on how to balance the application of community engagement to improve study participation and ethical research practice, with due recognition of underlying structural inequalities that may reinforce structural coercion and inhibit voluntary consent.

## Supplementary information

**Additional file 1.**

## Data Availability

The datasets used and/or analyzed during the current study are available from the corresponding author on reasonable request.

## Abbreviations

CIOMS: Council for International Organisations of Medical Sciences
FGD: Focus Group Discussions
HCW: Health Care Workers
IDI: In-depth interviews
US: United States of America
