# Supplementary material for: Structural coercion in the context of community engagement in global health research conducted in a low resource setting in Africa
Source: BMC Med Ethics. 2020 Sep 21;21:90. doi: 10.1186/s12910-020-00530-1 (PMC7504839; doi:10.1186/s12910-020-00530-1)
Supplement: Supplementary file 1 — Additional file 1. [file 12910_2020_530_MOESM1_ESM.docx]

**Topic guides document**

Table of Contents

[FGD topic guide v.2 140815 2](#_Toc48811491)

[Topic guide for research participants/non research participant V.2 5](#_Toc48811492)

[Topic guide for researchers/research staff V.2 7](#_Toc48811493)

[Topic guide for community leaders v.2 8](#_Toc48811494)

[Topic guide for community volunteers/CAG v. 2 9](#_Toc48811495)

**Engage study**

## **FGD topic guide v.2 140815**

**Section A: Engagement with health services**

1. Can you please tell me the names of institutions or organisations that work in this community?
   - What types of services does each of the provider offer?

**Section B: Engagement with researchers**

***Knowledge of health research***

1. Can you please tell me what you know about health research?

- Can you please tell me any recent research projects that are currently being done in this community that you are aware of? [Probe: Nature of research, target population]
- How did you know about these research projects?

1. What strategies were used to inform communities about research? **[Probe: Hospital, community]**
2. *Before the study*
3. *During study implementation*
4. *After implementing the study*

- How do people perceive the communication/ community engagement strategies that were being used to inform the community?
- How do people perceive the research procedures and ethical aspects of the study?
- Who makes decisions on engagement activity? What are community’s roles in the engagement activity?

***Community participation in research***

1. How do researchers work with communities? How is the community involved in research?

***[Probe: gatekeepers/ community members/ community representatives/ community groups/ Who are the field workers/ where do they come from?]***

- What do people think about research? To whom do you report concerns/challenges with research? How are they addressed? Any examples?
  - ***[Probe: gatekeepers/ community members/ community representatives/ community groups/ Who are the field workers/ where do they come from?]***
- What factors make people enrol in research?
- What factors make people refuse to enrol in a research? what factors make people withdraw from research?

***Benefits and risks***

1. What do people from this community expect from research? How are these expectations met?

- How do people look at researchers & research? (What are the perceived risks and benefits of research)?
- How has research benefited this community? How has it benefited individuals?
- What challenges have people experienced due to their involvement in research? How were these challenges resolved?

***Community representation***

1. Can you please tell me what you know about Community Advisory Groups/ community/research volunteers?

- How were they selected? How do you work with CAG members/ research volunteers?

***Perception of health services***

1. What services are available in your community If one suffers from ill health?
   1. **List down on a flip chart**
   2. [Probe: Private, government, traditional healers, religious healers]

- If someone is ill in your family and you don't go to western medical services, what alternative options are available in your community?
- What do you think about the quality of services offered at the places that we have listed down?
  - **(Personnel: staff attitudes, cost, distance, waiting time, availability of drugs, equipment)**
- What other issues affect your decisions about where to go If someone is sick?
  1. **Section C: *Relevance of current research to participating research communities***

1. Can you please tell me some of the issues that concern you as residents in this community?

***{Rank the concerns in order of priority from most significant to least significant concerns}***

- Why do these things concern you?
- What is currently being done to address some of the concerns that you have raised? Who or which institutions/ individuals are involved in addressing those concerns?
- What do you think should be done to address these concerns?

Thank you for your participation

**Engage study**

## **Topic guide for research participants/non research participant V.2**

**Experiences of research**

1. Before this research project, did you know about any health research project? what was the research about? how did you know about it?

- Can you please share any past experiences you have had in research? how about your close friends& family?

1. when was this?
2. where did it take place?
3. How did you/they hear about it?
4. What were you/ they expected to do?
5. What made you/them refuse or enrol in the study?
6. Did you/they have any concerns? How did you/they raise the concerns? How were they resolved?
7. How does this experience influence your perception of research/ researchers?

**Understanding of health research/ research ethics**

1. What is your understanding of health research? Research ethics? How did you know about this?

**Communication of research project**

1. How did you hear about the .....................study? (Probe: community/ hospital based sensitizations meetings, CAGs, research staff).
2. Who told you about it? Who brought the letters?
3. What did they tell you?

- How did you feel when you heard that your child/household/you were being invited in the research? Why did you feel that way?
- What did you think about the channel of communication/ information about the research? (Probe: personnel, relevance, effectiveness).
- What was important to you to know more about the research?
- Why was is important to know about that?
- What could be done to improve communication about the research? Why do you say so?

**Understanding of present study**

1. Can you please explain to me about the study that you are involved in?
2. Why it is being done?
3. What is it going to involve?
4. Who is going to be involved?
5. What are the benefits of this study?

**Reasons for enrolling in the study**

1. Why did you enrol in the research? Why did you refuse to enrol in the study? **(Probe: past experience, religion, rumours etc)**
2. How did you make the decision to participate*?*
3. What issues were considered, which of those mattered the most?
4. Who did you consult before you made the decision?
5. Why did you consult them?
6. What did they advise you?
7. How did other household members feel about your decision?
8. How did your friends/ neighbours feel about your decision?

- What other factors (If not mentioned) would make you enrol or refuse to enrol in a research? (Probe: **Information about study, Length of study, study procedures, rationale of study, incentives**)

**Expectations from research/community engagement**

1. What were/ are your expectations from the research?/ community engagement (Probe: personal benefits, public health benefits, feedback findings).

**Benefits and risks**

1. What were the benefits and risks of the research project?

- Do you have any concerns about the research?*?*
- Who did you share your concerns with? Has anyone shared their concerns about this research with you?
- How did they assist you?
- Why did you not share your concerns?
- What were some of the difficulties you faced while participating in the study? How were these challenges resolved? were you able to ask questions, seek more clarity or negotiate decisions with the researchers/ field workers? Why?
  - (Probe: **interaction with FWs, research procedure, context**)
- What views do you have with regards to the study?
- What was your understanding of your role in this research project?

***Community participation***

1. How do researchers involve community members or research participants in a research? How would you want to be involved in a research project? **(probe: decision making, advice**)

**Community Advisory Group/ research volunteers**

1. Are you aware of any group/ individuals who represent you to researchers? Community Advisory Group members/ research volunteers? How were they selected? How does the community work with CAGs

**Future** **participation in research**

1. In future If you are invited to participate in research, would you participate? Why or Why not? What would you consider in making that decision

***End***

**Engage study**

## **Topic guide for researchers/research staff V.2**

**Interview guide**

1. What is your understanding of community engagement?
2. Why are you engaging communities in your study? why is community engagement an important aspect of your study?
3. Can you please share with me your experiences of engaging communities in research?

(Probe: what strategies do you use? At what stage of the research do you use them? Who is involved in organising/ implementing community engagement? Who initiates it?)

1. How did you come up with the community engagement strategies? What is the rationale of each community engagement strategy mentioned? How does it link to the purpose of your community engagement plan?
2. How do you solicit/ address concerns from community? How do you use feedback from research participants during protocol development, implementation and presenting study findings?
3. What are your views/ perceptions of current practice/ community engagement strategies (Probe: cost, effectiveness, relevance, trust etc)
4. What are the benefits/ risks of your community engagement strategies? What makes it effective or ineffective? Give examples/ past experiences?
5. What successes/challenges have you experienced pertaining to community engagement?

(**Probe**: community understanding of research, consenting to participate

which aspects are they willing to participate, What can you comment in relation to community response across the different sites?

**Engage study**

## **Topic guide for community leaders v.2**

1. How many research projects are currently being implemented in this area?

- Hospital based vs community based
- Research topic
- Length of study

1. What is your understanding of health research?
2. How do researchers from this study work with you? How are you involved in research/engagement activities? What roles do you play?

- Before implementation
- During implementation
- After study

1. What are your expectations from community engagement/research?
2. What successes/challenges have you experienced with community engagement/research? How were the challenges resolved?
3. What are the risks and benefits of the community engagement activities/ research? How has community engagement/research benefited you? This community?
4. Are you aware of CAG/ community volunteers members? How do you work with CAG members/ community volunteers?

**Engage study**

## **Topic guide for community volunteers/CAG v. 2**

1. How were you selected as a research volunteer/CAG member?

- Who was involved in selecting you?
- Which chiefs were involved in selecting you?
- Why were you selected?
- When were you selected?

1. Which area are you responsible for?

- What's the name of the villages or chiefs?
- How big is your catchment location?
- How many households are in your catchment location?

1. How does a normal day look like to you?

- What activities are you involved in on a daily basis?
- What to do you do for a living?
- How do you manage your role as a research volunteer, your profession & other roles?

1. Which research projects are you aware of that are being implemented in this area?

- How did you know about these research projects?
- Can you tell more about each research project/ what is your role in all the research projects that you have mentioned?

1. What is your role in the ................research?

- What is your involvement in this research project?
- How do you engage with researchers?
- How do you engage with communities?
- What issues do community members often talk about?
- What do researchers expect from you? What do community members expect from you? what do you expect from researchers/research?

1. Can you tell me some of the successes in your work?

- What happened/ where/ who was involved?/when did this happen?

1. What challenges have you encountered in your work?
2. What motivated you to become a volunteer? Why have you remained a volunteer till this day?

*Thank you for your participation*
